# Nomogram model based on ultrasonography and contrast-enhanced CT for predicting BRAF^V600E^ mutation in thyroid nodules classified as C-TIRADS 3 and above

**DOI:** 10.3389/fendo.2025.1663456

**Published:** 2025-12-02

**Authors:** Wenran Zhang, Chenfan Yu, Yu Gao, Jiaqing Dou

**Affiliations:** Department of Endocrinology, Chaohu Hospital of Anhui Medical University, Hefei, China

**Keywords:** thyroid nodules, BRAF^V600E^ mutation, ultrasonography, contrast-enhanced CT, combined diagnosis, nomogram, multimodal

## Abstract

**Background:**

BRAF^V600E^ mutation detection enhances diagnostic accuracy in distinguishing benign from malignant thyroid nodules. This study aims to develop and validate a predictive model for the BRAF^V600E^ mutation in C-TIRADS 3 or higher nodules.

**Methods:**

A retrospective study was conducted involving 324 patients with C-TIRADS 3 or higher thyroid nodules. Based on BRAF^V600E^ testing from ultrasound-guided fine needle aspiration biopsy (FNAB), patients were divided into wild-type (n=263) and mutation(n=61) groups. Predictive features were independently selected from ultrasonography (US), contrast-enhanced CT (CECT), and combined imaging using Least Absolute Shrinkage and Selection Operator (LASSO) regression. Multivariate logistic regression analysis was employed to identify independent risk factors and then develop three predictive models. Model performance was evaluated through calibration curves, receiver operating characteristic (ROC) analysis, decision curve analysis (DCA), and Brier scores, respectively. The optimal model was subsequently converted into a visualized nomogram to facilitate clinical implementation.

**Results:**

Ultrasonographic microcalcifications were the strongest independent predictor of BRAF^V600E^ mutation (OR = 9.63, 95% CI: 3.62–25.63, P < 0.001). Higher C-TIRADS grades, irregular morphology on US, and blurred borders or capsule interruption on CECT were also significant independent risk factors. Notably, smaller nodule size on US correlated with higher mutation risk (OR = 0.93, 95% CI: 0.88–0.98, p=0.012). The multimodal model combining US and CECT (AUC = 0.937) outperformed individual US (AUC = 0.915) and CECT (AUC = 0.784) models.

**Conclusion:**

The nomogram integrating US and CECT features shows strong predictive performance and clinical utility for identifying BRAF^V600E^ mutations in C-TIRADS 3 or higher thyroid nodules.

## 1 Introduction

Thyroid nodules, defined as localized abnormal cellular proliferations within the thyroid gland, are typically detected through palpation or imaging examinations. While most nodules are benign and often incidentally discovered during routine physical examinations or US, clinically significant nodules may cause compression symptoms that impair normal swallowing or respiratory function when exceeding certain dimensions. In thyroid tissue, the BRAF mutation is most commonly defined by the V600E variant, caused by a T→A transversion at nucleotide 1799 (exon 15). Among malignant thyroid nodules, papillary thyroid carcinoma (PTC) represents the most prevalent histological subtype. Recent advances in molecular diagnostics have identified the BRAF^V600E^ mutation as a specific molecular marker for PTC (1, 2). The BRAF^V600E^ mutation results in the constitutive activation of its encoded oncoprotein, conferring elevated kinase activity. This leads to the aberrant and persistent activation of the MAPK signaling pathway (MEK1/2 and ERK1/2) through a phosphorylation cascade. The consequent dysregulated signaling serves as the principal molecular driver promoting tumorigenesis by inducing uncontrolled cellular proliferation, inhibiting apoptosis, and facilitating abnormal differentiation and invasion of thyroid cells (3–6). The BRAF^V600E^ mutation in thyroid nodules is a significant indicator of malignancy and is associated with greater biological aggressiveness (7, 8), manifesting as higher postoperative recurrence rates and poorer clinical prognosis. Current clinical guidelines advocate active surveillance for low-risk nodules. Conversely, BRAF^V600E^-positive cases warrant more aggressive interventions, such as surgical resection or enhanced monitoring protocols with more frequent US examinations. Precise assessment of BRAF mutation status is essential not only for definitive tumor diagnosis and effective anti-BRAF targeted therapy (e.g., vemurafenib and dabrafenib) but also for providing crucial prognostic information regarding the nodule’s biological behavior and potential aggressiveness (9, 10). While BRAF^V600E^ genotyping typically depends on surgical or fine-needle aspiration biopsy samples analyzed by RT-PCR or NGS, inadequate tissue or poor sample quality frequently impedes molecular diagnosis (11). Moreover, since a single biopsy or small tissue specimen only captures the genetic status of the sampled site, it fails to assess the comprehensive genetic panorama of the tumor, thereby potentially missing other critical co-mutations. Although droplet digital PCR has demonstrated high sensitivity and specificity in detecting BRAF mutations in plasma circulating cell-free DNA, its standalone diagnostic utility remains limited. In some cancer patients, the tumor burden may be insufficient to yield detectable levels of circulating tumor DNA (12). The development of imaging-based integrated models for predicting genetic mutation status, such as BRAF^V600E^, represents a highly promising direction in precision medicine. The core advantage of this approach lies in its ability to overcome the major limitations of conventional molecular detection methods. It is non-invasive, thereby avoiding the risks associated with additional biopsies, including bleeding, infection, and tumor seeding. Furthermore, by capturing information from the entire tumor lesion, it has the potential to provide a more comprehensive reflection of the tumor’s global biological behavior, thereby offering a novel perspective and tool for clinical decision-making.

The Thyroid Imaging Reporting and Data System (TI-RADS) is an ultrasound-based standardized classification system designed to stratify malignancy risk in thyroid nodules and guide clinical management. Thyroid nodules classified as TI-RADS categories 1 and 2 demonstrate minimal malignancy risk, obviating the need for fine-needle aspiration or routine surveillance. A progressive increase in malignant potential was observed with ascending TI-RADS classification levels (13). CECT is an imaging technique that utilizes intravenous contrast agents in conjunction with CT scanning to evaluate tissue vascularity and structural morphology. In the diagnostic workup of thyroid nodules, high-frequency US and CECT serve as complementary imaging modalities, each with distinct advantages and limitations. US, with its superior soft-tissue resolution, is the primary modality for evaluating internal nodular architecture - such as microcalcifications, solid components, and borders - as well as vascularity. Its real-time, dynamic, and radiation-free nature makes it ideal for initial screening, risk stratification, longitudinal follow-up, and, uniquely, for guiding fine-needle aspiration biopsy. However, its diagnostic accuracy is operator-dependent, and its utility is limited in assessing retrosternal extension, deep cervical lymph node metastases, and invasion into adjacent structures. In contrast, CECT provides a comprehensive anatomical roadmap of the neck and mediastinum. It excels at delineating the spatial relationships between a nodule and critical structures like the trachea, esophagus, and major blood vessels. This capability is indispensable for evaluating extra-thyroidal extension in advanced cancer, retrosternal growth, and mediastinal lymph node metastasis, thereby providing critical information for preoperative planning. Its principal limitation, however, lies in a lower sensitivity for detecting the subtle internal features of a nodule compared to US.

Most existing studies are confined to a single imaging modality, and their predictive performance has room for improvement. The combination of US and CECT offers a synergistic approach, enabling not only precise qualitative assessment and risk stratification of nodules but also a comprehensive evaluation of their overall extent and potential invasiveness. This facilitates a co-diagnostic process that integrates anatomical localization with pathological qualification, thereby optimizing therapeutic decision-making. Therefore, this study pioneers the construction of a comprehensive predictive model by integrating multimodal features from these two routine imaging techniques. We aim to explore the correlation between BRAF^V600E^ mutation status and multimodal imaging features, with the expectation that such informational complementarity will yield superior predictive performance compared to any single-modality approach. The primary objective of this study is to develop and validate multimodal predictive models for preoperatively assessing BRAF^V600E^ mutation status. We will construct and compare three distinct models: one based on US features, another on CECT features, and an integrated model combining both. Through a comparative performance analysis, the optimal model will be selected to establish a risk stratification tool. The ultimate goal is to provide clinicians with a more reliable diagnostic framework to facilitate timely intervention for thyroid nodules with a high risk potential.

## 2 Materials and methods

### 2.1 Patient selection

This retrospective study enrolled 324 patients with thyroid nodules classified as TI-RADS category 3 or higher, who were diagnosed and treated at our hospital’s Department of Endocrinology between October 2022 and April 2024. The cohort comprised 66 male and 258 female patients, with a mean age of 51.25 ± 12.46 (range 16–77) years and a mean body mass index (BMI) of 24.33 ± 3.47 kg/m². All participants underwent ultrasound-guided FNAB of the thyroid. Based on the BRAF^V600E^ gene status assessed in the fine-needle aspiration (FNA) samples, patients were stratified into two groups: a wild-type group (n=263) and a mutant group (n=61).

**Figure 1:**
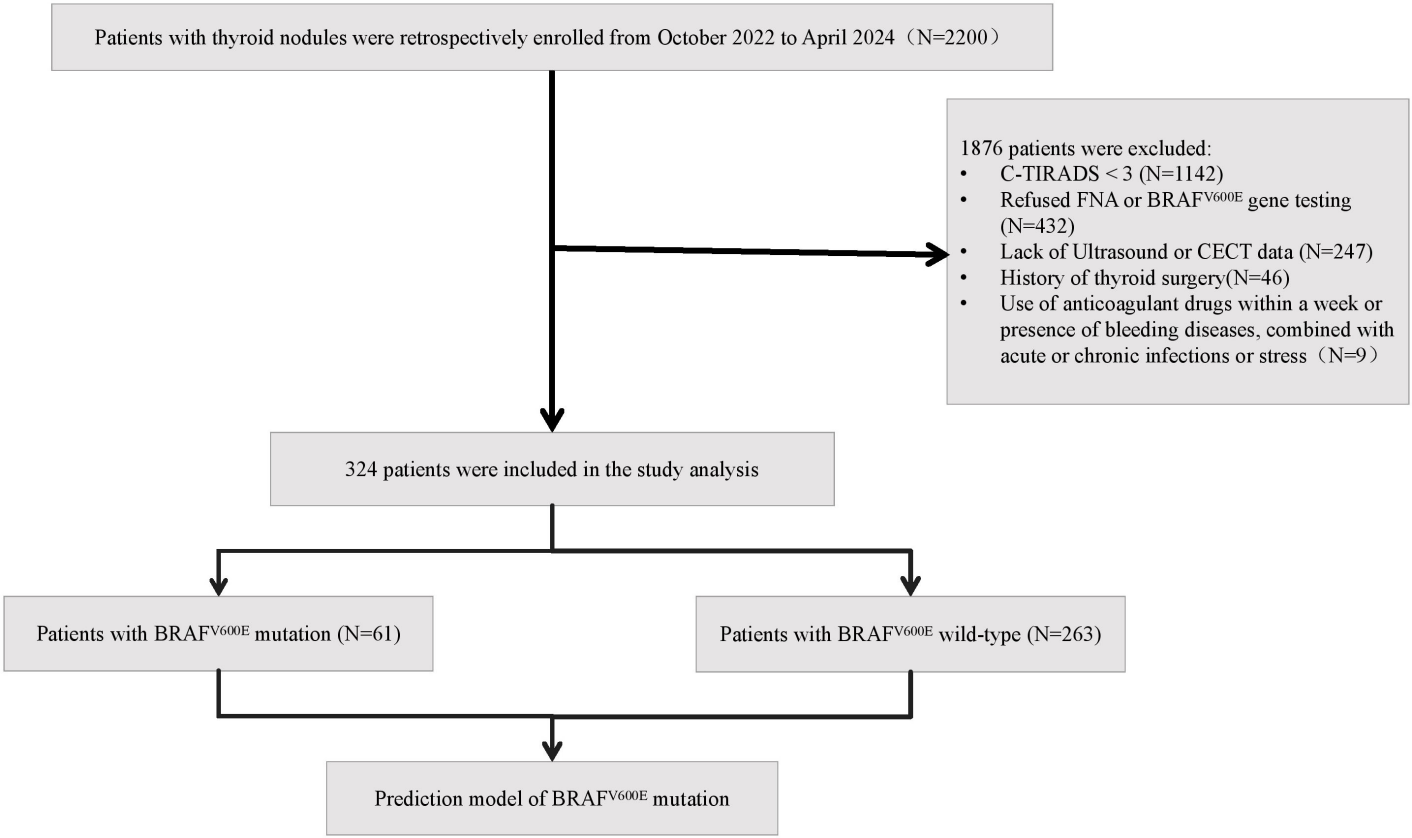
Flow chart of study population enrollment.

Inclusion criteria comprised: (a) ultrasonographically confirmed TI-RADS category 3 or higher nodules; (b) completion of both ultrasound-guided FNAB with BRAF^V600E^ genetic testing; and (c) availability of complete US and CECT examination results. Exclusion criteria included: (a) anticoagulant use within one week or presence of bleeding disorders (e.g., hemophilia); (b) concurrent acute/chronic infections or stress conditions; (c) history of previous thyroid surgery; and (d) incomplete clinical data. The flow chart of study population enrollment is detailed in **Figure 1**. The study was reviewed and approved by the Medical Ethics Committee of Chaohu Hospital Affiliated with Anhui Medical University (Approval no. KYXM-202209-006).

### 2.2 Clinical data collection

Demographic and clinical characteristics, including age, sex, and BMI, were retrospectively collected from the hospital’s electronic medical record system.

### 2.3 Ultrasonography protocol

All patients underwent standardized thyroid US using a Samsung HERA W9 color Doppler system with high-frequency linear array transducers (5–12 MHz). The following sonographic features were documented: C-TIRADS category, nodule diameter (mm), echogenicity (hypoechoic or non-hypoechoic), composition (solid or non-solid), multifocality (single nodule or multiple nodules), margin (well-defined or ill-defined), any visible cervical lymph nodes (invisible or visible), capsule (intact or interrupted), morphology (regular or irregular), calcifications (non-calcification, microcalcification or macrocalcification), echotexture (homogeneous or heterogeneous), and color Doppler flow imaging (CDFI) findings (avascular or hypervascular). Each nodule was systematically evaluated in both transverse and longitudinal planes by at least two experienced radiologists blinded to the clinical data. To assess the objectivity of feature interpretation, inter-observer agreement was calculated using the independent assessments made by the two physicians before consensus discussion. Cohen’s kappa was computed for categorical variables, weighted kappa for ordinal categorical variables, and the intraclass correlation coefficient (ICC) for continuous variables. All agreement coefficients exceeded 0.7, indicating good consistency. In cases of discrepant assessments, the two radiologists re-evaluated the images jointly to reach a consensus, and the agreed-upon results were used for subsequent statistical analysis.

### 2.4 Contrast-enhanced CT protocol

CECT examinations were performed using the GE MEDICAL SYSTEMS Light-Speed 64-line spiral CT scanner. The imaging protocol included non-contrast, arterial, and venous phases: a bolus of iodixanol (60–85 mL) was administered via the antecubital vein at an injection rate of 2–2.8 mL/s, followed by enhanced scanning at 25 s (arterial phase) and 60 s (venous phase) after injection. The scanning range extended from the mastoid process to the sternal notch, with a reconstructed slice thickness of 1–3 mm. Two radiologists independently evaluated the following CECT characteristics, including nodule density (even or uneven), boundary (clear or blurred), detection of any visible cervical lymph nodes (invisible or visible), calcifications (yes or no), and capsule (intact/interrupted). Inter-observer agreement was assessed using the kappa statistic based on the two radiologists’ independent evaluations before consensus discussion. All agreement coefficients exceeded 0.7, indicating good consistency. In cases of discrepant assessments, the radiologists re-evaluated the images jointly to reach a consensus, and these consensus results were used for subsequent statistical analysis.

### 2.5 BRAF^V600E^ genetic testing and pathological examination protocol

Samples obtained from thyroid nodules via ultrasound-guided FNA were analyzed for BRAF^V600E^ mutation. DNA was extracted using a micro-pathological nucleic acid extraction kit (Roche Ltd.), followed by real-time quantitative PCR analysis on the SLAN-96S system. Fluorescence intensity was quantified to calculate Ct and ΔCt values using reference standards. A result was defined as positive when the FAM channel Ct value was <38 and ΔCt <9; samples not meeting both criteria were recorded as negative. This validated threshold was established through extensive testing on cell lines and clinical samples with known mutation status to ensure optimal specificity and sensitivity. The pathological diagnoses were established based on FNAB for non-surgical patients and postoperative paraffin-embedded section results for surgical patients.

### 2.6 Statistical analysis

Statistical analyses were performed using SPSS 27.0, R 4.4.1 and GraphPad Prism 10.1.2., with normally distributed continuous variables expressed as mean ± standard deviation (x̄ ± s) and compared using independent samples t-tests, while non-normally distributed data were presented as median (P25, P75) and analyzed using Mann-Whitney U tests; categorical variables were described as frequencies or percentages and compared using chi-square or Fisher’s exact tests. Using BRAF^V600E^ mutation status as the dependent variable, potential predictors were initially screened via LASSO regression. Variables selected by LASSO were subsequently incorporated into binary logistic regression to identify independent predictors and construct a BRAF^V600E^ mutation risk prediction model. Model stability was internally validated using 1000 bootstrap resamples, with performance assessed through calibration curves, receiver operating characteristic (ROC) curves, decision curve analysis (DCA), and the Brier score. The following R packages were employed: rms(v7.0-0), car(v3.1-3), survival(v3.8-3), pROC(v1.18.5), tcltk(v4.4.1), ResourceSelection(v0.3-6), DescTools(v0.99.60), rmda(v1.6), Rcpp(v1.0.14).

## 3 Results

### 3.1 Comparative analysis of baseline characteristics and imaging features between groups

To investigate the relationship between genetic mutation status and the clinicopathological/imaging features of thyroid nodules, we conducted a comparative analysis between the mutant and wild-type groups (**Table 1**). The results revealed significant differences in both baseline characteristics and multiple imaging features between the two groups. Regarding baseline characteristics, patients in the mutant group were significantly younger (median age: 46 years) than those in the wild-type group (median age: 54 years). A significant difference in gender distribution was also observed, with a higher proportion of males in the mutant group (34.4% vs. 17.1%, P = 0.002). No statistically significant difference in BMI was found between the groups (P = 0.053).

**Table 1:** Comparative analysis of baseline characteristics and imaging features between groups.

Characteristics	Wild type	Mutant type	Z/t/x^2^	P
Age	54 (47,59)	46 (33,57)	-3.272	0.001
Gender			9.153	0.002
Female	218(82.9)	40(65.6)		
Male	45(17.1)	21(34.4)		
BMI	24.15 ± 3.44	25.10 ± 3.53	-1.939	0.053
Ultrasonography				
C-TIRADS			85.037	<0.001
3	184(70.0)	6(9.8)		
4a	66(25.1)	40(65.6)		
4b	12(4.6)	9(14.8)		
4c	1(0.4)	5(8.2)		
5	0(0.0)	1(1.6)		
Size	16 (9,27)	9 (5,14)	-5.877	<0.001
Echogenicity			20.109	<0.001
Non-hypoechoic	100(38.0)	5(8.2)		
Hypoechoic	163(62.0)	56(91.8)		
Composition			10.985	<0.001
Non-solid	69(26.2)	4(6.6)		
Solid	194(73.8)	57(93.4)		
Multifocality			3.753	0.053
Single	63(24.0)	22(36.1)		
Multiple	200(76.0)	39(63.9)		
Margin			3.827	0.050
Well-defined	232(88.2)	48(78.7)		
Ill-defined	31(11.8)	13(21.3)		
Lymph node			21.262	<0.001
Invisible	206(78.3)	30(49.2)		
Visible	57(21.7)	31(50.8)		
Capsule			4.625	0.032
Intact	258(98.1)	56(91.8)		
Interrupted	5(1.9)	5(8.2)		
Morphology			26.156	<0.001
Regular	234(89.0)	38(62.3)		
Irregular	29(11.0)	23(37.7)		
Calcification			111.762	<0.001
Non-calcification	218(82.9)	20(32.8)		
Microcalcification	11(4.2)	34(55.7)		
Macrocalcification	34(12.9)	7(11.5)		
Echotexture			2.559	0.110
Homogeneous	134(51.0)	38(62.3)		
Heterogeneous	129(49.0)	23(37.7)		
CDFI			4.621	0.032
Avascular	179(68.1)	50(82.0)		
Hypervascular	84(31.9)	11(18.0)		
Contrast-Enhanced CT				
Density			2.027	0.155
Even	200(76.0)	41(67.2)		
Uneven	63(24.0)	20(32.8)		
Boundary			24.797	<0.001
Clear	131(49.8)	9(14.8)		
Blurred	132(50.2)	52(85.2)		
Lymph node			10.944	<0.001
Invisible	135(51.3)	17(27.9)		
Visible	128(48.7)	44(72.1)		
Calcification			3.123	0.077
NO	216(82.1)	44(72.1)		
Yes	47(17.9)	17(27.9)		
Capsule			48.980	<0.001
Intact	248(94.3)	38(62.3)		
Interrupted	15(5.7)	23(37.7)		
Pathological diagnosis			124.304	<0.001
Benign nodules	223(84.8)	8(13.1)		
PTC	40(15.2)	53(86.9)		

In terms of ultrasonographic features, nodules in the mutant group were smaller in size and had higher C-TIRADS risk classifications. While the majority of wild-type nodules were categorized as C-TIRADS 3 (70.0%), most mutant-type nodules were classified as C-TIRADS 4a or higher (90.2%). Additionally, mutant-type nodules more frequently presented with a combination of hypoechoicity, solid composition, cervical lymph nodes, interrupted capsules, and irregular morphology. A particularly notable difference was observed in calcification patterns (P < 0.001), with microcalcifications being substantially more common in the mutant group (55.7%) compared to the wild-type group (4.2%). Interestingly, the mutant group showed a lower prevalence of hypervascularity on color Doppler flow imaging (P = 0.032). Margin demonstrated a borderline association with BRAF^V600E^ genotype (P = 0.050), with ill-defined margins showing a trend toward higher frequency in the mutant group (21.3% vs. 11.8%). No significant differences were found in thyroid echotexture or multifocality between the two groups.

CECT characteristics similarly revealed significant differences, with the mutant group more frequently demonstrating blurred boundaries (85.2% vs. 50.2%, P < 0.001), interrupted capsules (37.7% vs. 5.7%, P < 0.001), and detectable cervical lymph nodes (72.1% vs. 48.7%, P < 0.001). No statistically significant differences were observed in post-contrast density homogeneity or the presence of calcification.

Furthermore, BRAF^V600E^ mutation showed a strong association with malignant pathological diagnoses (P < 0.001). The mutation rate was significantly higher in papillary thyroid carcinoma (53/93, 57.0%) compared to benign nodules (8/231, 3.5%).

### 3.2 Feature selection using LASSO regression for US, CECT, and combined models

**Figure 2:**
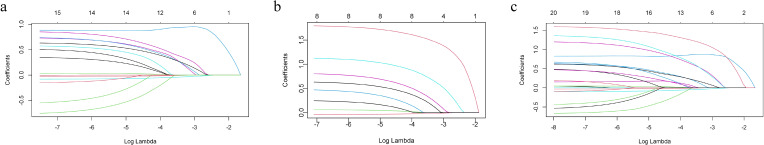
Path plots of LASSO regression coefficients for the three models. The x-axis represents log-lambda, reflecting the degree of regularization. As the lambda value increases, the coefficient values shrink gradually to zero, and the number of retained variables decreases. **(a)** US model. **(b)** CECT model. **(c)** Multimodal model of US combined with CECT.

**Figure 3:**
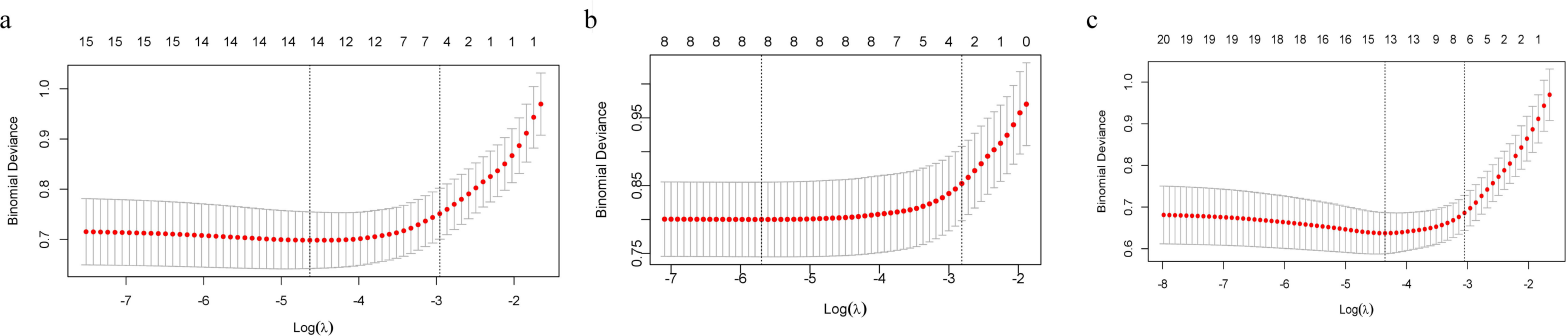
LASSO regression cross-validation curves for the three models. The x-axis is log-lambda; the upper axis denotes the number of non-zero coefficients, while the left y-axis represents the binomial deviance. The graph demonstrates the variation in binomial deviance with different lambda values. The two vertical lines represent the optimal lambda values selected by cross-validation. The left dashed line is lambda.min (the value that minimizes the binomial deviance), and the right dashed line is lambda.1se (the most regularized model within one standard error of the minimum deviance). **(a)** US model. **(b)** CECT model. **(c)** Multimodal model of US combined with CECT.

This study employed LASSO regression to comprehensively evaluate the predictive value of clinical and imaging parameters for BRAF^V600E^ mutational status, which served as the dependent variable. The independent variables encompassed three categories: demographic characteristics (age, sex, BMI), ultrasonographic features (C-TIRADS category, nodule size, echogenicity, composition, multifocality, margin, lymph node, capsule, morphology, calcification, echotexture, and CDFI), and CECT characteristics (nodule density, boundary, lymph node, calcification, and capsule). The prediction model constructed solely on ultrasonographic features identified five significant predictors with non-zero coefficients at a penalty parameter lambda of 0.052 (lambda 1se): C-TIRADS category, nodule size, the presence of lymph nodes, morphology, and calcification. In contrast, the model based on CECT selected three key predictors at lambda. 1se = 0.060, including age, boundary, and capsule. Furthermore, a multimodal integrated model was developed by combining both ultrasonographic and CECT features. This combined model ultimately identified six optimal predictive indicators at lambda. 1se = 0.047, namely C-TIRADS category, nodule size, morphology, and calcification from US, alongside boundary and capsule from CECT. **Figure 2** displays the path plots of LASSO regression coefficients for the three models. **Figure 3** presents the LASSO regression cross-validation curves for the three models.

### 3.3 Multivariate logistic regression analysis of BRAF^V600E^ mutation predictors

Subsequent multivariate logistic regression analysis was performed on the six optimal variables selected by the combined US and CECT model to identify predictors independently associated with the BRAF^V600E^ mutation status in thyroid nodules (**Table 2**). Due to the limited sample size of C-TIRADS 4c and 5 nodules, they were merged with category 4b into a ‘4b+’ group for analysis. The results identified microcalcifications on US as the strongest independent predictor of the mutation (OR = 9.63, 95% CI: 3.62–25.63, P < 0.001). In contrast, macrocalcifications showed no significant statistical association with mutation risk (P = 0.627). Furthermore, several other features were established as significant independent risk factors. These included the higher C-TIRADS grade on US (4b+ vs. 3: OR = 7.75, 95% CI: 1.98–30.27, P = 0.003; 4a vs. 3: OR = 4.39, 95% CI: 1.54–12.53, P = 0.006), the irregular morphology (OR = 2.80, 95% CI: 1.09–7.25, P = 0.033), the blurred boundary on CECT (OR = 3.21, 95% CI: 1.23–8.37, P = 0.017), and the interrupted capsule on CECT (OR = 4.71, 95% CI: 1.71–12.97, P = 0.003). Notably, nodule size demonstrated a negative association with BRAF^V600E^ mutation risk (OR = 0.93, 95% CI: 0.88–0.98, P = 0.012), indicating that, within this model, smaller nodules were associated with a higher probability of harboring the mutation.

**Table 2:** Multivariate logistic regression analysis of predictors for the BRAF^V600E^ mutation in C-TIRADS ≥3 thyroid nodules.

Characteristics	β	Standard error	Wald	P	OR(95%CI)
C-TIRADS (US)			10.375	0.006	
4a	1.480	0.535	7.663	0.006	4.393(1.540-12.527)
4b+	2.047	0.695	8.673	0.003	7.748(1.983-30.266)
Size (US)	-0.071	0.028	6.342	0.012	0.932(0.882-0.984)
Morphology (US)	1.031	0.485	4.528	0.033	2.804(1.085-7.248)
Calcification (US)			21.121	<0.001	
Microcalcification	2.265	0.499	20.587	<0.001	9.633(3.621-25.626)
Macrocalcification	0.270	0.555	0.236	0.627	1.310(0.442-3.884)
Boundary (CECT)	1.168	0.488	5.716	0.017	3.214(1.234-8.370)
Capsule (CECT)	1.550	0.517	8.992	0.003	4.710(1.711-12.969)

Reference categories: C-TIRADS 3 for C-TIRADS grading; regular for morphology; non-calcification for calcification; clear for boundary; intact for capsule.

### 3.4 Model performance comparison

#### 3.4.1 Calibration curves

**Figure 4:**
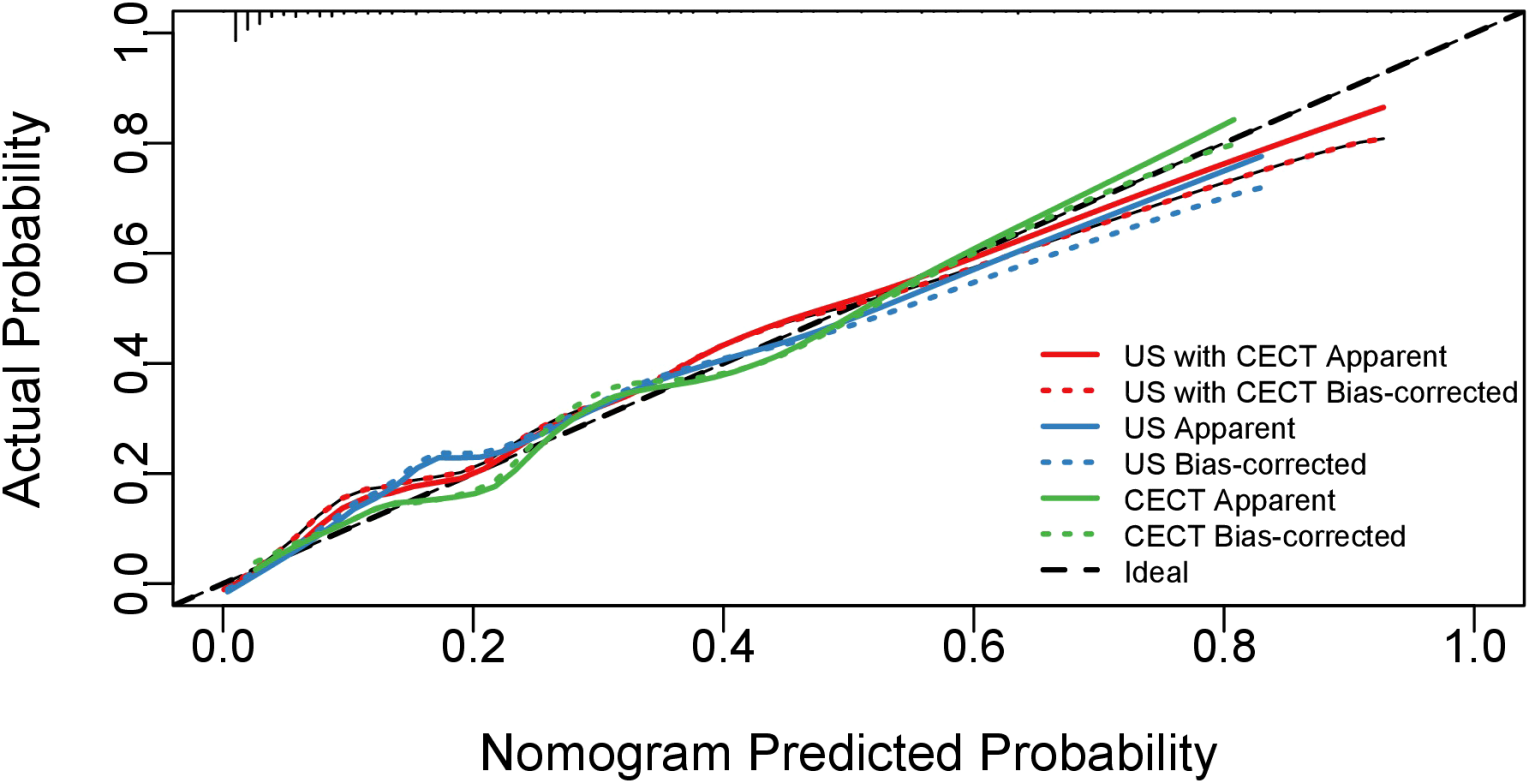
Calibration curves of the three models. The diagonal dotted line represents the ideal prediction of a perfect model. The solid lines depict the performance of various models, where the ‘Apparent’ curves represent the apparent predictive accuracy, and the ‘Bias-corrected’ curves represent the validated performance after bias correction. The closer a solid line is to the ideal line, the better the model’s calibration.

The model performance evaluation using bootstrap resampling with 1000 iterations for internal validation demonstrated that all models passed the Hosmer-Lemeshow goodness-of-fit test (P > 0.05). Comparative analysis revealed that the combined US and CECT model exhibited optimal calibration performance, with its calibration curve demonstrating the closest approximation to the ideal reference line. Quantitative evaluation of calibration indices further confirmed that this integrated model had the minimal calibration error among all tested models. Specifically, the combined model showed excellent agreement between predicted and observed outcomes (χ²=6.59, P = 0.58), outperforming both the CECT-alone model (χ²=11.96, P = 0.15) and the US-alone model (χ²=10.97, P = 0.20). These comparative results are visually presented in **Figure 4**.

#### 3.4.2 ROC curves analysis

**Figure 5:**
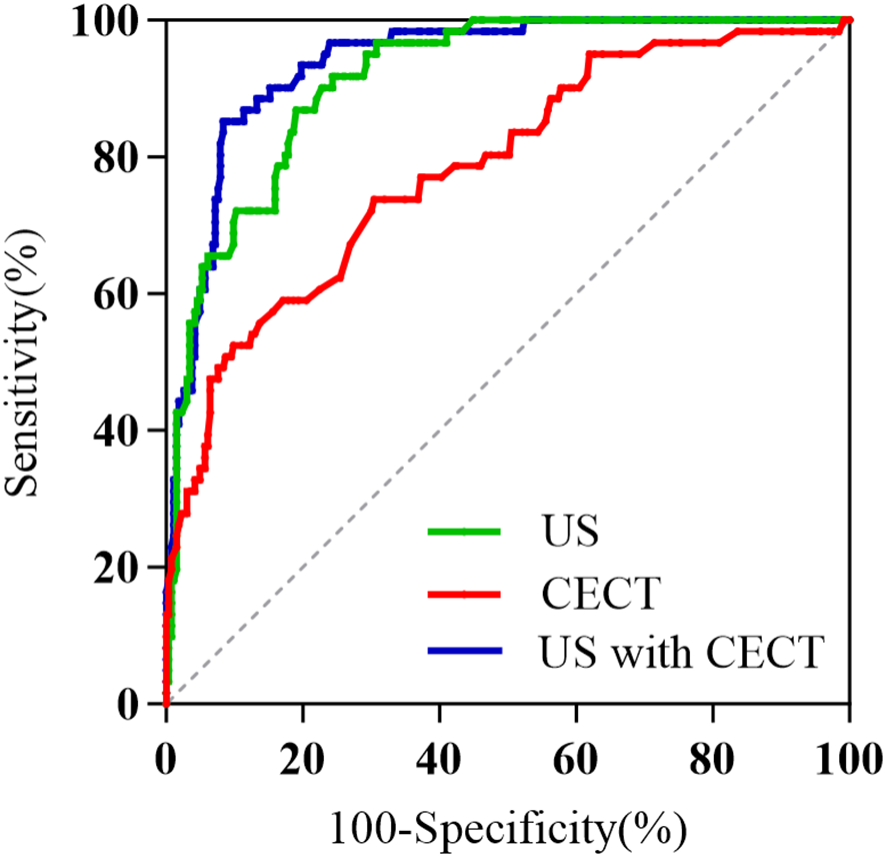
ROC curves of the three models. The area under the curve (AUC) serves as an indicator of predictive accuracy and generalization capability.

ROC curve analysis demonstrated that the multimodal diagnostic model integrating US and CECT exhibited significantly superior discriminatory performance (AUC = 0.937) compared to the US-alone model (AUC = 0.915) or the CECT-alone model (AUC = 0.784). At the optimal cutoff value, the combined model showed excellent diagnostic performance, with a sensitivity of 85.2%, specificity of 91.6%, and accuracy of 90.4%. Furthermore, the positive predictive value (PPV) and negative predictive value (NPV) were 70.3% and 96.4%, respectively, indicating its exceptional value in ruling out negative diagnoses (**Table 3**, **Figure 5**). Internal validation was performed using the bootstrap method with 1,000 resampling iterations. The mean AUC from this validation was 0.905 (95% CI: 0.894–0.911), with a standard deviation of 0.004. This result, being close to the original estimate, confirms the model’s good discriminative ability. In summary, the integrated model demonstrates favorable and reliable performance in distinguishing BRAF^V600E^ mutation status.

**Table 3:** Comparison of the predictive performance of different models for BRAF^V600E^ gene mutations in TI-RADS category 3 and above.

Model	AUC (95%CI)	P	Sensitivity	Specificity	Accuracy	PPV	NPV
US	0.915(0.881-0.948)	<0.001	0.869	0.810	0.824	0.519	0.968
CECT	0.784(0.718-0.850)	<0.001	0.738	0.696	0.704	0.360	0.920
Multimodal	0.937(0.909-0.966)	<0.001	0.852	0.916	0.904	0.703	0.964

#### 3.4.3 Decision curve analysis

**Figure 6:**
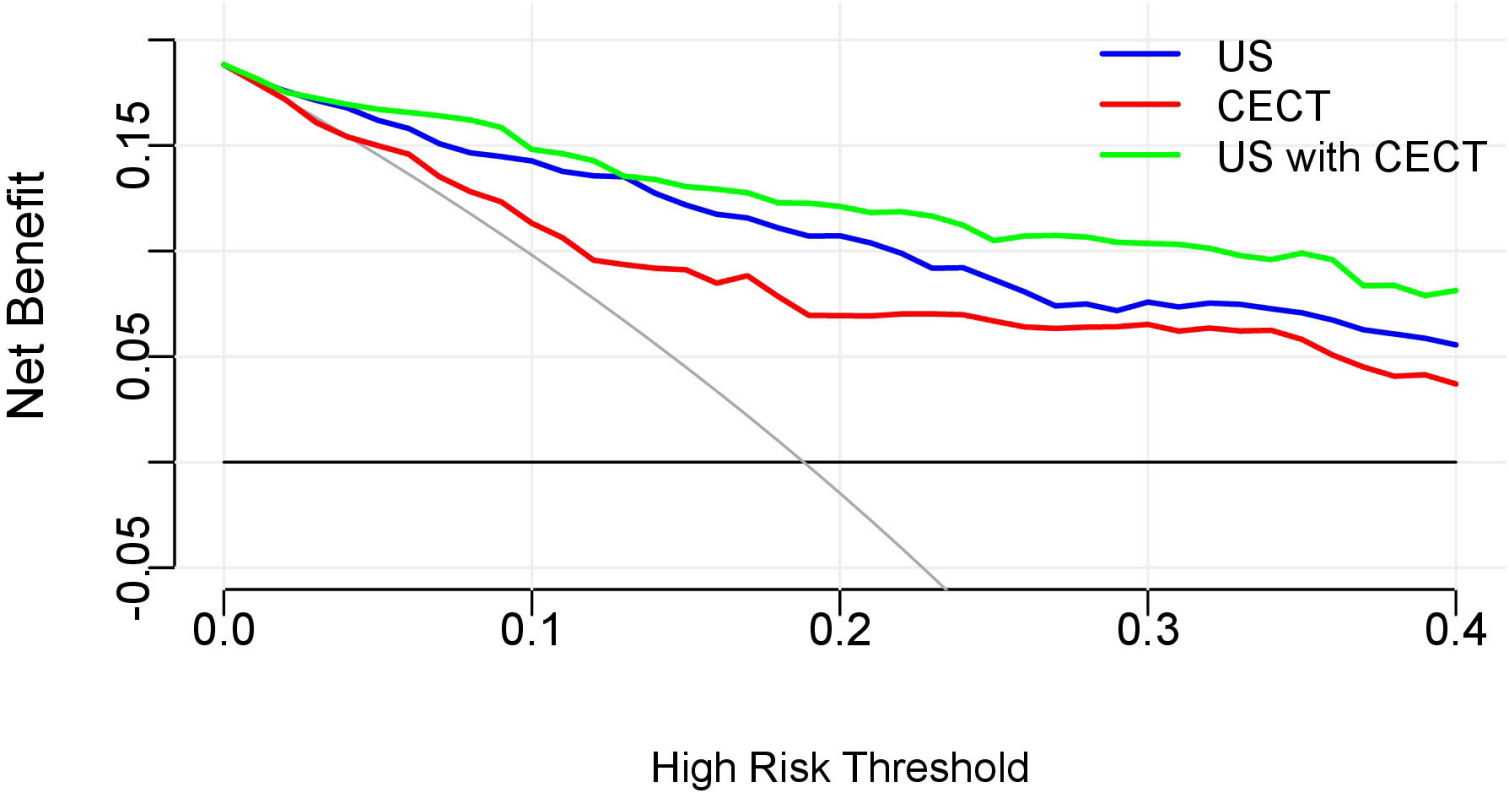
Decision curves analysis of the three models. The x-axis of the curve represents the risk threshold probability, and the y-axis represents the net clinical benefit derived from decisions based on the prediction model. The colored curves indicate the net benefits obtained by applying different nomogram models, respectively. The gray curve represents the net benefit of the ‘treat all’ strategy, while the black curve represents the net benefit of the ‘treat none’ strategy.

Decision curve analysis was performed to evaluate and compare the clinical utility of three distinct prediction models: the US-alone model, the CECT-alone model, and the combined US-CECT multimodal model. The analysis plotted net clinical benefit (y-axis) against the continuum of risk threshold probabilities (x-axis), enabling quantification of each model’s clinical value across different decision-making scenarios. The decision curve analysis revealed significant differences in net benefit among the three models across threshold probabilities (**Figure 6**). The combined US-CECT model demonstrated superior clinical utility in the low-risk threshold range (10-40%), achieving the highest net benefit and suggesting optimal performance for intermediate-risk clinical decision-making. As predicted by decision curve theory, all models exhibited progressively decreasing net benefits at higher threshold probabilities. The integrated US and CECT model demonstrated consistently superior net benefit across clinically relevant threshold probabilities, outperforming single-modality approaches.

#### 3.4.4 The Brier scores

In the comprehensive evaluation of predictive model performance using 1000 bootstrap resampling iterations, the combined US and CECT model demonstrated significantly lower prediction error (Brier score = 0.091) compared to both the US-alone model (Brier score =0.106, P = 0.026) and CECT-alone model (Brier score = 0.118, P<0.001), indicating superior overall accuracy of the multimodal approach. No statistically significant difference was observed between the single-modality models (US vs CECT: P = 0.242), suggesting comparable predictive accuracies. Thus, the combined model ultimately proved to be the most reliable tool for BRAF^V600E^ mutation risk stratification in thyroid nodules.

### 3.5 Nomogram of US combined with CECT model

**Figure 7:**
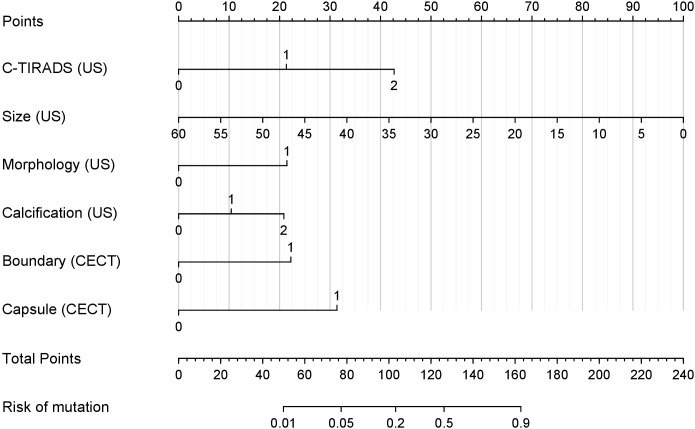
Nomogram of the risk of BRAF^V600E^ mutation in thyroid nodules of TI-RADS class 3 and above. The nomogram integrates multiple predictors to estimate the probability of BRAF^V600E^ mutation risk in thyroid nodules classified as C-TIRADS 3 or above. Each variable is assigned a point value based on its contribution to the model. The total points (sum of individual variable points) correspond to the predicted mutation risk at the bottom scale.

Based on the results of the LASSO and multivariate logistic regression analyses, we constructed a nomogram (**Figure 7**) that integrates US and CECT features to facilitate individualized prediction of malignancy risk in thyroid nodules. The model incorporates six key predictors: C-TIRADS (US), size (US), morphology (US), calcification (US), boundary (CECT), and capsule (CECT). This nomogram provides a visual interface that transforms the complex regression model into a convenient scoring system, thereby enabling a quantitative estimation of malignancy probability.

The nomogram is applied by referring to the ‘Points’ axis for each variable to determine the individual score based on the patient’s features. After summing these scores, the total is located on the ‘Total Points’ axis. Projecting downward to the ‘Risk of Mutation’ axis provides the estimated personalized malignancy probability. Illustrative Example: A nodule with a C-TIRADS 4a classification (21 points), a size of 25 mm (58 points), the regular morphology (0 points), and the presence of microcalcifications (21 points) is assessed by US. On CECT, it demonstrates a clear boundary (0 points) and an intact capsule (0 points). The summation yields a total of 100 points, corresponding to a predicted mutation risk of approximately 20%.

## 4 Discussion

Medical imaging serves as a well-established, non-invasive diagnostic tool, offering a promising avenue for predicting genomic status. Previous studies have validated the efficacy of CT-based radiomic models in predicting KRAS, NRAS, and BRAF mutations in colorectal cancer (14–16). In the context of thyroid cancer, CT radiomics has been primarily applied to assess the risk of lymph node metastasis (17). However, compared to its successful application in the genotyping of colorectal cancer, the potential of CT radiomics for predicting key driver gene mutations in thyroid cancer, such as BRAF^V600E^, remains underexplored. Concurrently, while ultrasonographic radiomics has demonstrated well-established value in differentiating benign from malignant thyroid nodules (18), its utility in predicting BRAF^V600E^ mutation has been limited (19, 20). Furthermore, radiomic analysis typically requires specialized software and additional computational time, preventing its seamless integration into existing clinical workflows and thus posing challenges for practical application in routine clinical settings. Therefore, the BRAF^V600E^ mutation prediction model developed in this study, which integrates conventional US and CECT features, offers distinct advantages over previous radiomics models. These advantages lie not only in a potential improvement in predictive accuracy but, more importantly, in its innovative multimodal approach. This integration enables complementary strengths of the two imaging modalities, thereby significantly enhancing the model’s interpretability and its acceptability to clinicians.

The core clinical value of the US combined CECT nomogram model lies in its provision of a novel decision-support tool for the refined and individualized management of thyroid nodules classified as C-TIRADS 3 and above. In terms of clinical applicability, all variables incorporated into the model are routinely and readily available from standard clinical examinations, requiring no additional costly or invasive procedures. This ensures the model’s strong generalizability and operational feasibility in diverse practice settings. The potential impact of this model on clinical decision-making is twofold. First, for nodules predicted to be at high risk of BRAF^V600E^ mutation, clinicians can recommend preoperative fine-needle aspiration for genetic testing or consider diagnostic surgery with greater confidence. This approach aids in mitigating the risk of underdiagnosis and delayed treatment for high-risk lesions. Second, for nodules predicted to be at low risk, the model serves as a valuable supplement to the conventional C-TIRADS classification. It provides additional evidence to support the choice of ‘active surveillance’ over immediate biopsy, thereby potentially reducing unnecessary invasive procedures along with their associated complications and healthcare costs. For clinicians, this model offers a powerful decision-making aid. It translates complex imaging features into an intuitive probability of mutation risk. When faced with nodules of diagnostic uncertainty, it empowers physicians to make more informed assessments regarding the benefits and risks of biopsy, facilitating the development of tailored management strategies. Ultimately, this tool is poised to enhance the overall precision and confidence in the clinical management of thyroid nodules.

Multiple studies have indicated a significant association between the BRAF^V600E^ mutation and specific ultrasonographic features (21–23). Consistent with these reports, our study identified a higher C-TIRADS category, an irregular morphology, and the presence of microcalcifications as independent risk factors for the BRAF^V600E^ mutation in thyroid nodules. Conversely, nodule size emerged as an independent protective factor, with smaller nodules exhibiting a higher mutational risk. These findings suggest that the BRAF^V600E^ mutation does not occur randomly but is closely associated with a more aggressive imaging phenotype. The underlying mechanism likely involves the malignant biological behavior driven by this mutation, which directly manifests as these characteristic sonographic changes (24). Our study demonstrated that the risk of BRAF^V600E^ mutation increases significantly with ascending C-TIRADS categories. This result aligns with previous research, which has also reported significant correlations between the BRAF^V600E^ mutation and higher risk scores in both EU-TIRADS and ACR TI-RADS systems (25, 26). Collectively, this converging evidence from different TIRADS frameworks confirms a strong link between the BRAF^V600E^ mutation and imaging features indicative of malignancy. It underscores the universal value of standardized sonographic categorization in the preoperative, noninvasive prediction of this pivotal molecular event. An irregular nodular morphology is a recognized sonographic marker of invasive growth behavior in thyroid cancer, which is closely linked to molecular pathways driven by the BRAF^V600E^ mutation. Consequently, a strong and well-defined positive correlation exists between these two features. In malignant nodules, excessively rapid tumor cell proliferation can lead to the overgrowth of fibrous tissue and blood vessels, subsequently causing calcium salt deposition and the formation of calcifications. In our study, only microcalcifications demonstrated clinical value, whereas macrocalcifications showed no statistical significance. This discrepancy may be attributed to the fact that macrocalcifications often result from dystrophic calcification within fibrotic connective tissue, which is not typically associated with malignant or invasive potential (27). The relationship between nodule size and malignancy risk remains controversial. Some studies suggest that smaller nodules (e.g., those with a diameter < 2.0 cm) may harbor a higher probability of malignancy (28, 29). For instance, Kamran et al. reported that the malignancy risk increased proportionally in nodules measuring 1.0–2.0 cm but stabilized beyond this threshold (30). In contrast, other perspectives maintain that nodule size alone is not a reliable predictor of malignancy risk (31). Our study revealed that smaller nodules were associated with a higher risk of BRAF^V600E^ mutation. This finding implies that the BRAF^V600E^ mutation may be present from the early stages of tumorigenesis, acting as an initial driver event that confers malignant potential even in small nodules (30). As a nodule enlarges, cytological heterogeneity may develop, with malignant cells coexisting with a substantial proportion of benign cells, potentially diluting the mutational signal (32). Additionally, selection bias in clinical detection could influence these observations. Smaller nodules are often selected for genetic testing or surgery due to suspicious cytological or imaging features, thereby increasing the sensitivity of mutation detection. In contrast, larger nodules are frequently resected due to compressive symptoms or cosmetic concerns, even when their cytology is benign. This difference in clinical pathways might lead to a relatively higher false-negative rate for mutation detection in larger nodules (33). In summary, no unified consensus currently exists regarding the association between nodule size and BRAF^V600E^ mutation or overall thyroid cancer risk. This complex relationship involves intricate tumor biological mechanisms and clinical detection biases, warranting further investigation for clarification.

An analysis of medical records from over one million patients revealed a positive correlation between the utilization of neck CT imaging and an increased incidence of thyroid cancer across different population subgroups (34). In line with the heightened diagnostic sensitivity of imaging, our study found that thyroid nodules exhibiting blurred boundaries and interrupted capsules on CECT were significantly associated with the BRAF^V600E^ mutation. This distinct imaging phenotype is not incidental but rather a macroscopic reflection of the specific tumor biology driven by the BRAF^V600E^ mutation. Mutated tumor cells secrete a variety of proteases that degrade the extracellular matrix, facilitating invasive growth into the surrounding thyroid parenchyma. Concurrently, this process induces the formation of aberrantly abundant neovessels at the tumor periphery (35). On CECT, these pathological changes manifest as an enhancing, invasive rim, collectively contributing to the ‘blurred boundaries’. When these highly aggressive mutant tumors expand, they directly invade and breach the thyroid capsule. This pattern stands in sharp contrast to the expansive growth typically observed in benign nodules. Even when significantly enlarged, benign nodules are often surrounded by a fibrous capsule, maintaining a clear demarcation from adjacent tissues (36).

The multimodal model developed from the aforementioned imaging features holds significant value for predicting BRAF^V600E^ mutation risk in thyroid nodules categorized as C-TIRADS 3 and above. Compared to models based solely on the US or CECT, the integrated model demonstrated superior performance across all evaluation metrics: its calibration curve was closest to the ideal line, it achieved the highest AUC, it yielded a higher net benefit across a wider range of threshold probabilities in decision curve analysis, and it had the lowest Brier score. These results collectively indicate optimal performance in calibration, discrimination, clinical utility, and overall accuracy, underscoring the considerable promise of fusing US and CECT features for preoperative, non-invasive genotyping.

A key consideration in this study was the inclusion of TI-RADS category 3 nodules. We recognize that current guidelines classify these nodules as having a low malignancy risk and typically recommend active surveillance rather than immediate intervention. The primary objective of our prediction model is not to overturn this standard management strategy but to address a more nuanced clinical scenario. Specifically, it is intended to aid in decision-making when the management of certain TI-RADS 3 nodules—for instance, those that are large (e.g., >2 cm), cause patient anxiety, or exhibit growth during follow-up—is being reconsidered for potential FNA. At this critical juncture, our model integrates ultrasonographic and CECT features to provide a probability assessment for the presence of the high-risk BRAF^V600E^ mutation. A high-risk prediction from the model could offer evidence-based support for BRAF mutation testing in such nodules, potentially leading to earlier definitive diagnosis and intervention, thereby avoiding possible diagnostic delays. Conversely, a low-risk prediction could reinforce clinician and patient confidence in continuing with active surveillance. Future studies should focus on validating the clinical utility and cost-effectiveness of this model within this specific TI-RADS category 3 subgroup for guiding precision FNA and genetic testing strategies.

This study has several limitations that should be acknowledged. First, the sample size was relatively limited, with a moderate class imbalance between the BRAF^V600E^ mutant (n = 61) and wild-type (n = 263) groups. This imbalance may introduce selection bias and potentially affect the model’s predictive performance. Despite initial efforts to mitigate class imbalance via oversampling and undersampling, both methods resulted in models with compromised performance relative to the model developed from the unaltered data (**Supplementary Materials 1**-**3**). Given these findings, data resampling was therefore abandoned in the final analytical approach. To mitigate the impact of class imbalance, we employed several strategies: we prioritized the retention of features strongly associated with the mutation, applied L1 regularization for feature selection to eliminate redundant variables, and implemented a weighted logistic regression approach that assigned a higher misclassification cost to samples in the mutant group. This encouraged the model to focus more on learning from the minority class during fitting. These measures were taken to objectively reflect the model’s performance across both nodule types. Second, this study has a single-center, retrospective design. All enrolled cases were recruited from a single institution, and patients who declined FNA or BRAF^V600E^ genetic testing were excluded, which may introduce selection bias. Furthermore, the model’s performance may be influenced by our center-specific patient demographics, imaging protocols, and interpretation conventions, potentially limiting the generalizability of the developed nomogram. To address these limitations, we are currently expanding the sample size at our center and have initiated a collaborative multi-center study with other peer-level teaching hospitals in the region. We aim to establish a larger, more balanced cohort to facilitate external validation, thereby further verifying the reliability and generalizability of our findings. Finally, the FNA procedure is subject to sampling variability. The accuracy of BRAF^V600E^ genotyping may be compromised by false-negative results, which can arise from intratumoral heterogeneity or inadequate sampling of the mutational component.

## 5 Conclusion

In conclusion, the multimodal imaging model based on US and CECT has better predictive performance in predicting BRAF^V600E^ gene mutations of thyroid nodules of TI-RADS category 3 and above compared with the single imaging feature model. This validated multimodal model could potentially reduce costs by improving risk stratification and reducing clinically unnecessary genetic testing. In addition, clinical decision support can be enhanced by individualized estimates of the probability of malignancy that can inform diagnosis and treatment pathways. It is beneficial to optimize the allocation of resources in the medical system while maintaining the accuracy of diagnosis.

## Data Availability

The raw data supporting the conclusions of this article will be made available by the authors, without undue reservation.

## Supplementary material

Supplementary Material 1 Comparative Visualization of Data Distributions Before and After Resampling. The chart displays the class distribution of the original imbalanced data alongside the balanced distributions resulting from SMOTE oversampling and random undersampling.

Supplementary Material 2 Comparison of Model Performance Metrics Between the Original Dataset and Resampling Techniques (SMOTE and Undersampling).

Supplementary Material 3 Comparative Heatmap of Model Performance Across Different Data Processing Strategies. This heatmap visually compares six performance metrics (rows) evaluated on three datasets (columns). Color intensity corresponds to the score value, with darker hues indicating better model performance.
