# Supplementary figures and images for: Nomogram model based on ultrasonography and contrast-enhanced CT for predicting BRAFV600E mutation in thyroid nodules classified as C-TIRADS 3 and above
Source: Front Endocrinol (Lausanne). 2025 Dec 2;16:1663456. doi: 10.3389/fendo.2025.1663456 (PMC12705410; doi:10.3389/fendo.2025.1663456)

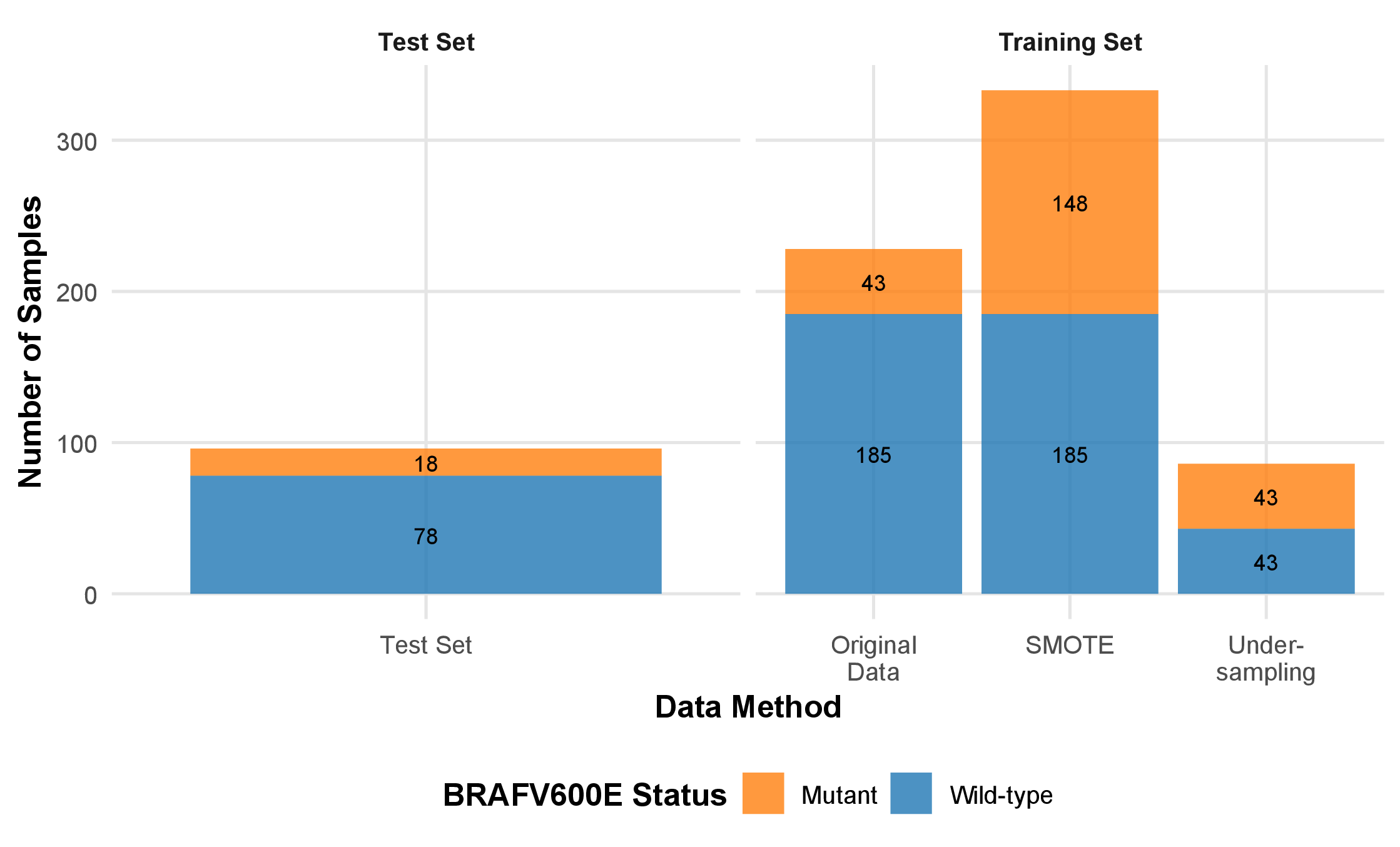

Supplement: Supplementary Material 1 — Comparative Visualization of Data Distributions Before and After Resampling. The chart displays the class distribution of the original imbalanced data alongside the balanced distributions resulting from SMOTE oversampling and random undersampling. [file DataSheet1.zip › Supplementary Materials/S1.tiff]

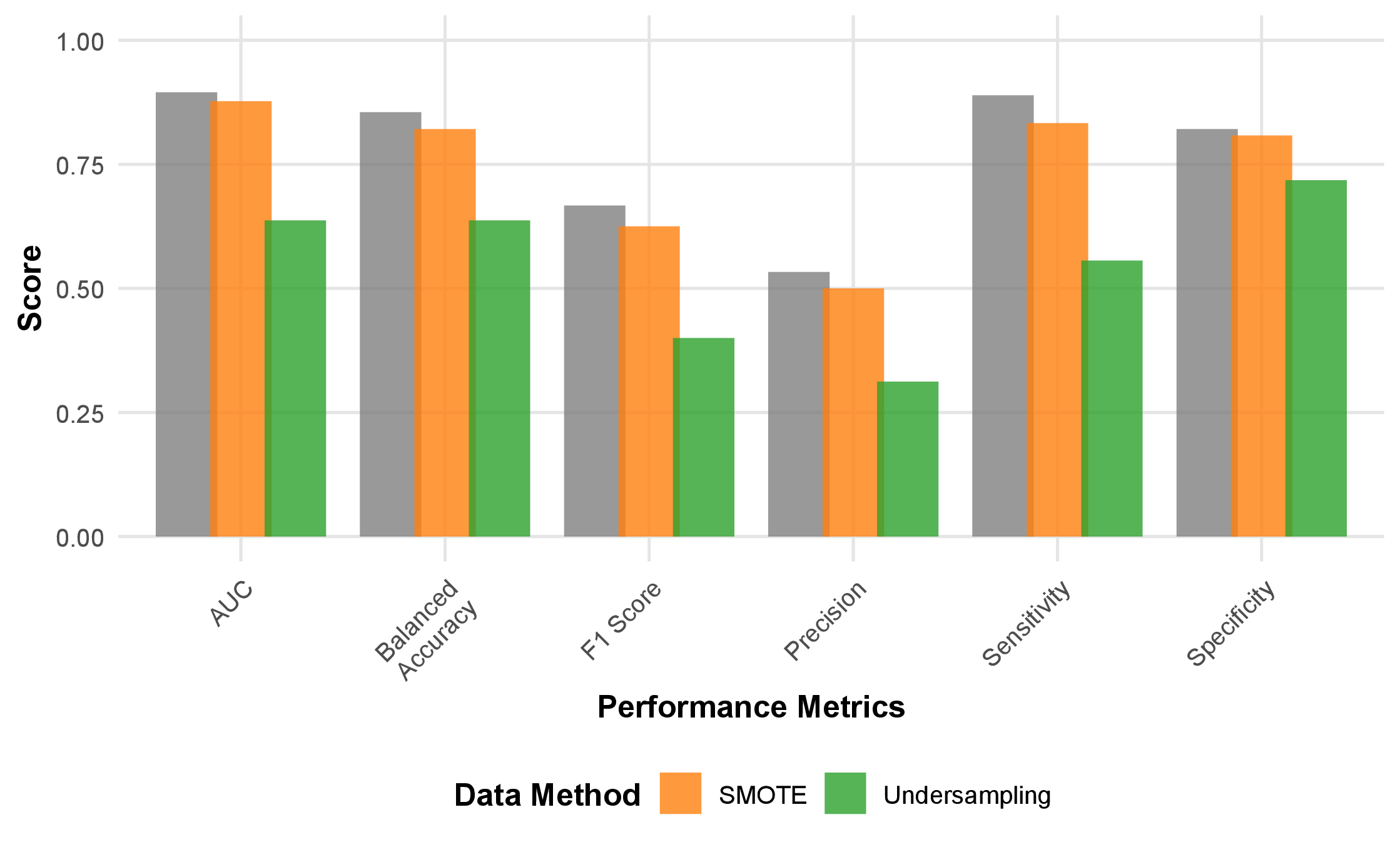

Supplement: Supplementary Material 1 — Comparative Visualization of Data Distributions Before and After Resampling. The chart displays the class distribution of the original imbalanced data alongside the balanced distributions resulting from SMOTE oversampling and random undersampling. [file DataSheet1.zip › Supplementary Materials/S2.tiff]

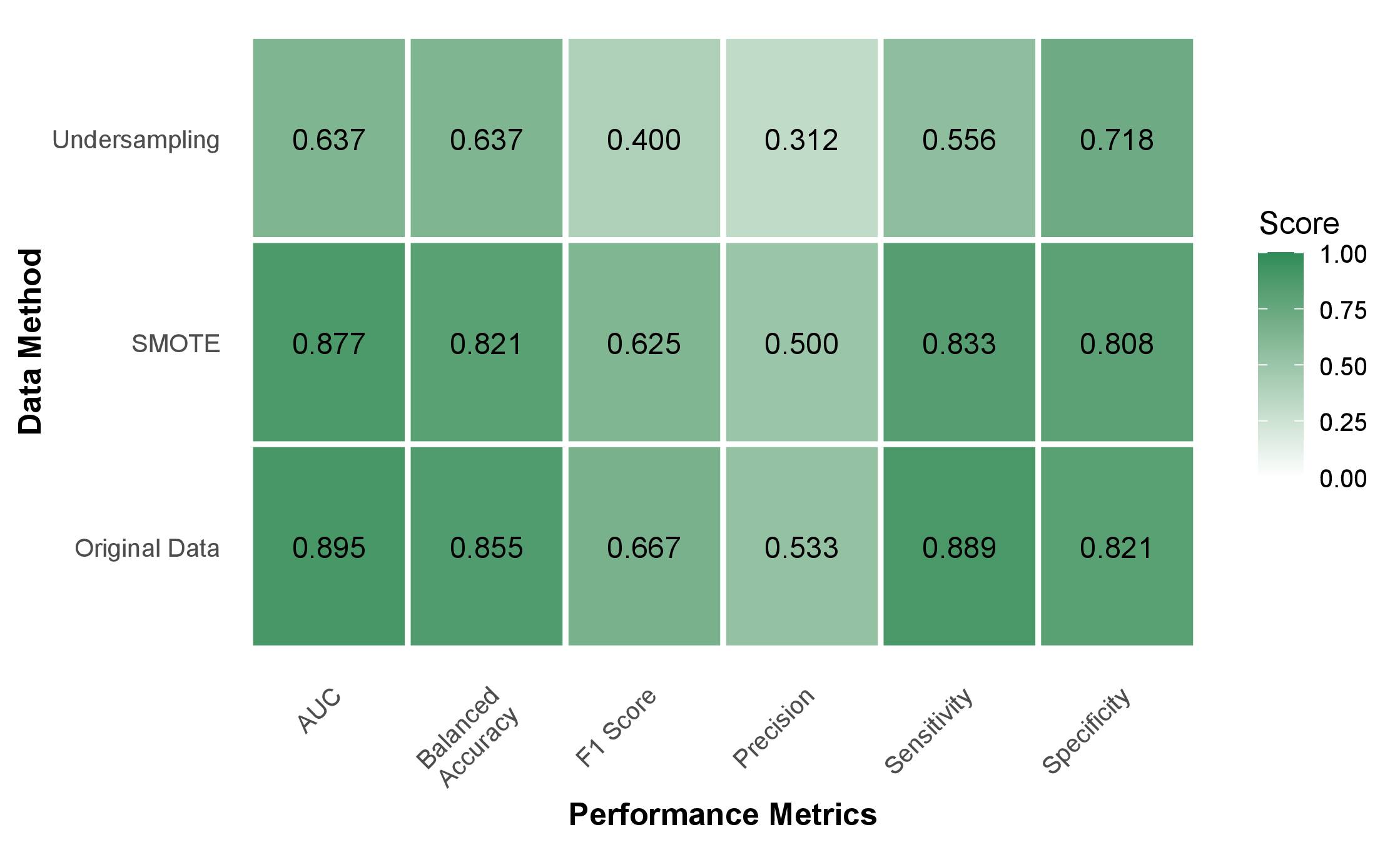

Supplement: Supplementary Material 1 — Comparative Visualization of Data Distributions Before and After Resampling. The chart displays the class distribution of the original imbalanced data alongside the balanced distributions resulting from SMOTE oversampling and random undersampling. [file DataSheet1.zip › Supplementary Materials/S3.tiff]
